# Different methods of methyl eugenol application enhance the mating success of male Oriental fruit fly (Dipera: Tephritidae)

**DOI:** 10.1038/s41598-018-24518-5

**Published:** 2018-04-16

**Authors:** Ihsan ul Haq, Carlos Cáceres, José S. Meza, Jorge Hendrichs, Marc J. B. Vreysen

**Affiliations:** 1 Insect Pest Control Laboratory, Joint FAO/IAEA Division of Nuclear Techniques in Food and Agriculture, Seibersdorf, Austria; 2 National Agricultural Research Centre, Park Road, Islamabad, Pakistan; 3 Insect Pest Control Section, Joint FAO/IAEA Division of Nuclear Techniques in Food and Agriculture, Vienna, Austria

## Abstract

Males of *Bactrocera dorsalis* (Hendel) (Diptera: Tephritidae) are strongly attracted to methyl eugenol (ME) (1,2-dimethoxy-4-(2-propenyl)benzene), a phenylpropanoid compound occurring in many plant species. Feeding on ME is known to enhance male *B*. *dorsalis* mating competitiveness, which can increase the effectiveness of the sterile insect technique (SIT) manifold. However, currently used systems for holding the mass-reared males in fly emergence and release facilities before release, do not allow for application of ME through feeding. Therefore, the current study was designed to evaluate different delivery systems of ME that would be applicable for large-scale application to sterile males held in such facilities. Males of a genetic sexing strain (GSS) of *B*. *dorsalis* treated by ME-aromatherapy or ME-airblown-aromatherapy that were competing with ME-fed males achieved a similar level of mating success in walk-in field cages, but the mating success was significantly higher when compared to untreated males. The results confirm the feasibility of developing ME-airblown-aromatherapy as a practical way of large scale ME delivery to enhance the mating competitiveness of sterile *B*. *dorsalis* males.

## Introduction

The Oriental fruit fly *Bactrocera dorsalis* (Hendel) (Diptera: Tephritidae), is native to tropical Asia and has invaded and become established over much of sub-Saharan Africa and the Pacific Islands, and its incursions are often intercepted by plant protection services in the USA and other countries^1,2^. *Bactrocera dorsalis* is widely recognized as either one of the most damaging horticultural pests where it occurs or as a high level quarantine threat to fruit production of importing countries where it is absent but capable of invasion and establishment^2,3^. *Bactrocera dorsalis* males, like many other *Bactrocera* species, are strongly attracted to methyl eugenol (ME) (1,2-dimethoxy-4-(2-propenyl) benzene), a phenylpropanoid compound naturally occurring in many plant species^2,4–6^. Since the identification of ME as a male attractant for certain *Bactrocera* species^7^, this chemical has been used for fruit fly population monitoring and as part of an environmentally-friendly, lure-and-kill approach termed male annihilation technique (MAT)^8–10^. The MAT has been successfully used to eradicate outbreaks or isolated established populations of *B*. *dorsalis* from the Marianas Islands, Micronesia^11^, Okinawa Islands, Japan^12^, Mauritius^13^ and Easter Islands and various *Bactrocera* spp. from California and Florida (USA)^14,15^.

The sterile insect technique (SIT), which is likewise an environmentally benign technique, involves the mass-rearing of male insects, sterilizing them by ionizing radiation, and releasing them over the target area in numbers large enough to outcompete their wild counterparts^16^. Wild females that mate with sterile males produce no offspring, and therefore, the systematic release of sterile males in adequate numbers reduces the wild population. The SIT becomes more effective when applied after suppressing the wild population^17,18^. Integration of the MAT with the SIT has so far been sequential with the SIT applied after a significant reduction of the wild population with the MAT^11,19,20^ to avoid killing too many of the released sterile males in ME-baited devices/traps, which would significantly reduce the efficacy of the SIT. *Bactrocera dorsalis* males once fed on ME showed two to three times less tendency of revisiting the ME-baited devices/traps than non-ME fed males in laboratory and field experiments^21^. A recent study on the Queensland fruit fly *Bactrocera tryoni* (Froggatt) male’s response to lure also showed that lower number of raspberry ketone (RK) fed males were recaptured in Cue-Lure (CL: the acetate of RK) baited traps than RK deprived males under both semi natural and field experiments^22^. Therefore, there is a potential for simultaneously implementing the SIT and the MAT^23,24^. Furthermore, feeding on ME was shown to enhance the mating competitiveness of *B*. *dorsalis* males as compared with males that had not been exposed to ME^25^.

Despite the effect of ME on male mating performance across *Bactrocera* species^26–28^ and its potential to increase the cost-effectiveness of the SIT, its operational use in fly emergence and release facilities has so far been limited due to the lack of a logistically practicable system to deliver ME to mass-reared sterile males before their release in the field. The common methods used for large-scale emergence and holding of sterile fruit flies prior to field releases^29–31^ do not allow the feeding of ME to millions of adult flies after their emergence. Additionally, in view of its toxicity, exposure to ME must be brief^32^. Tan and Tan^33^ designed a machine to feed ME to sterile males, where males are brushed off and collected after feeding ME from the ME-impregnated conveyer belt. While this innovative system works well under experimental conditions, treating millions of sterile males per day on an industrial scale will be challenging, if not impossible. Therefore, there is a need to develop simple methods of exposing sterile males to ME in fly emergence and release facilities that are compatible with current emergence and holding conditions.

The system that is routinely used in Mediterranean fruit fly *Ceratitis capitata* (Wiedemann) SIT programmes to increase mating success of sterile males^29,34^ consists of blowing a mist of volatiles of ginger root oil (GRO) into the sterile male holding rooms. This method of ‘aromatherapy’ allows sexually mature males to obtain access to GRO through condensation, inhalation, or impregnation with the chemicals in the circulating air prior to their release in the field^35–37^. ‘Aromatherapy’ was first used by Shelly *et al*.^36^ and is now an accepted concept and terminology for GRO application of Mediterranean fruit fly males. The uptake of ME is different than for GRO and occurs via ‘pharmacophagy’. Therefore, it was assumed that only ME ingestion could enhance the male’s mating success by producing more attractive pheromones for females. However, *Bactrocera carambolae* (Drew and Hancock) males exposed to ME volatiles achieved higher mating success, justifying the use of the term ‘aromatherapy’^38^.

In view that *B*. *dorsalis* has a much wider distribution than *B*. *carambolae* and is a pest of very high economic and quarantine importance, a method of ME delivery which can enhance male’s mating success and adoptable in sterile males release facilities would increase the cost effectiveness of SIT application manifold in area-wide integrated pest management programmes (AW-IPM) against *B*. *dorsalis*. Here we show that a modified method ‘ME-airblown-aromatherapy’ is more practical for implementation, than feeding, in sterile male emergence and release facilities. Further, we show that the selected method increases male mating competitiveness to a level equivalent to, or better than ME-fed, ME-aromatherapy, or untreated males.

## Results

**Figure 1:**
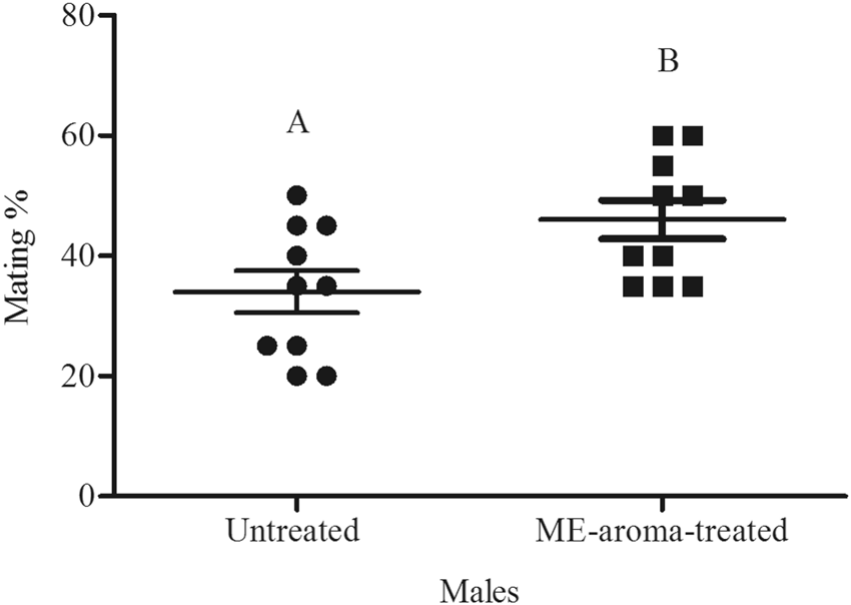
Mean mating percentage of ME-aroma-treated and untreated *Bactrocera dorsalis* males. The age of males on commencement of mating test was 15 d and they were treated with ME 1 d before mating test. Symbols represent raw data for 10 replicates; horizontal lines represent mean ± SE. Mean male mating success followed by different letters are significantly different from each other (Student’s *t*-test, *P* < 0.05).

**Figure 2:**
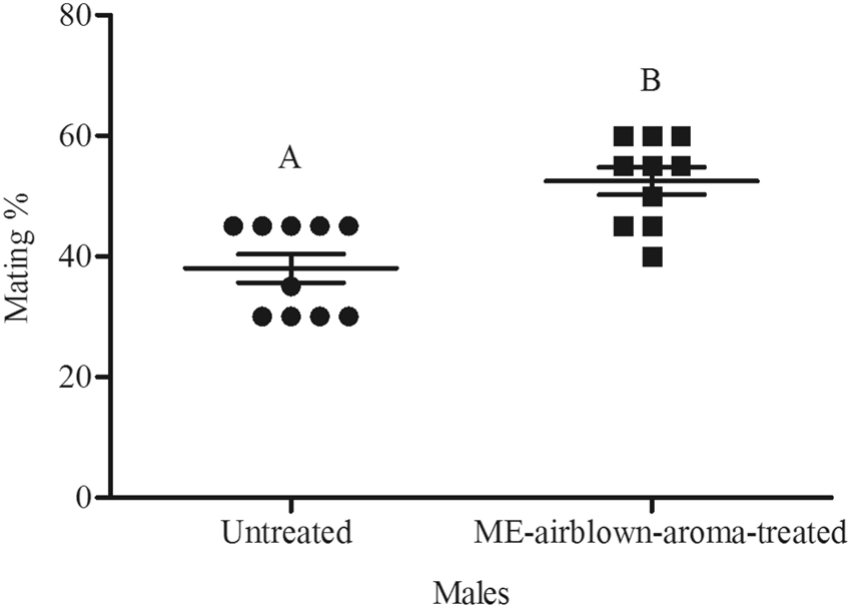
Mean mating percentage of ME-airblown-aroma-treated and untreated *Bactrocera dorsalis* males. The age of males on commencement of mating test was 15 d and they were treated with ME 2 d before mating test. Symbols represent raw data for 10 replicates; horizontal lines represent mean ± SE. Mean male mating success followed by different letters are significantly different from each other (Student’s *t*-test, *P* < 0.05).

**Figure 3:**
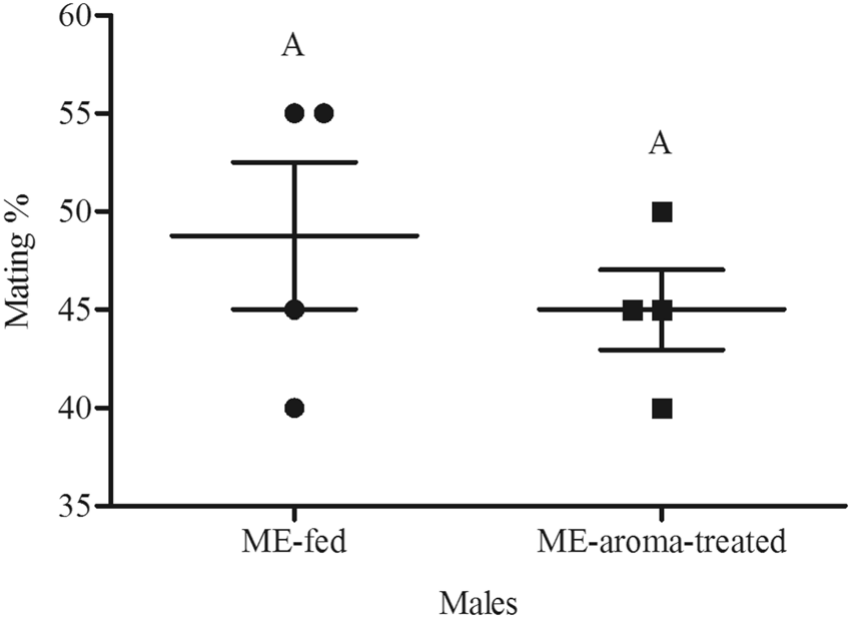
Mean mating percentage of ME-fed and ME-aroma-treated *Bactrocera dorsalis* males. The age of males on commencement of mating test was 17 d and they were treated with ME 2 d before mating test. Symbols represent raw data for 4 replicates; horizontal lines represent mean ± SE. Mean male mating success followed by different letters are significantly different from each other (Student’s *t*-test, *P* < 0.05).

**Figure 4:**
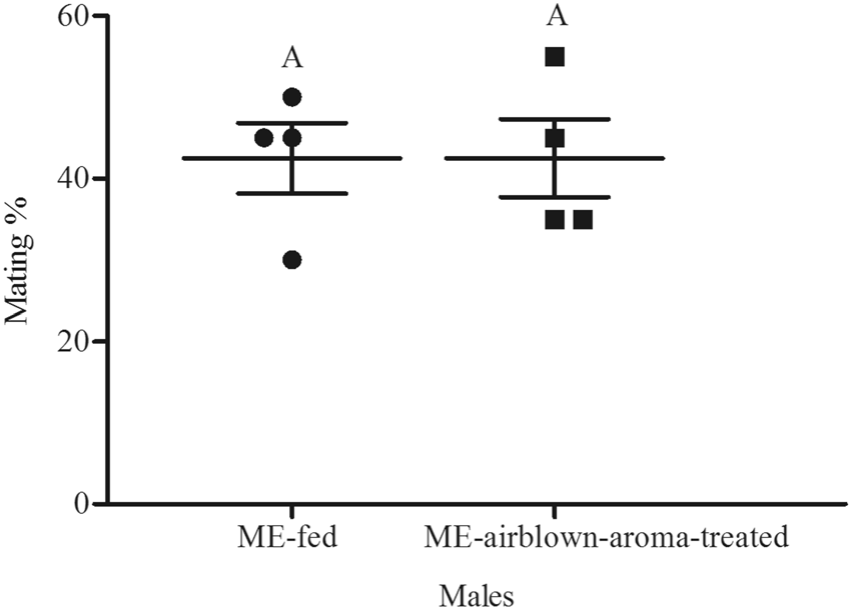
Mean mating percentage of ME-fed and ME-airblown-aroma-treated *Bactrocera dorsalis* males. The age of males on commencement of mating test was 15 d and they were treated with ME 2 d before mating test. Symbols represent raw data for 4 replicates; horizontal lines represent mean ± SE. Mean male mating success followed by different letters are significantly different from each other (Student’s *t*-test, *P* < 0.05).

**Figure 5:**
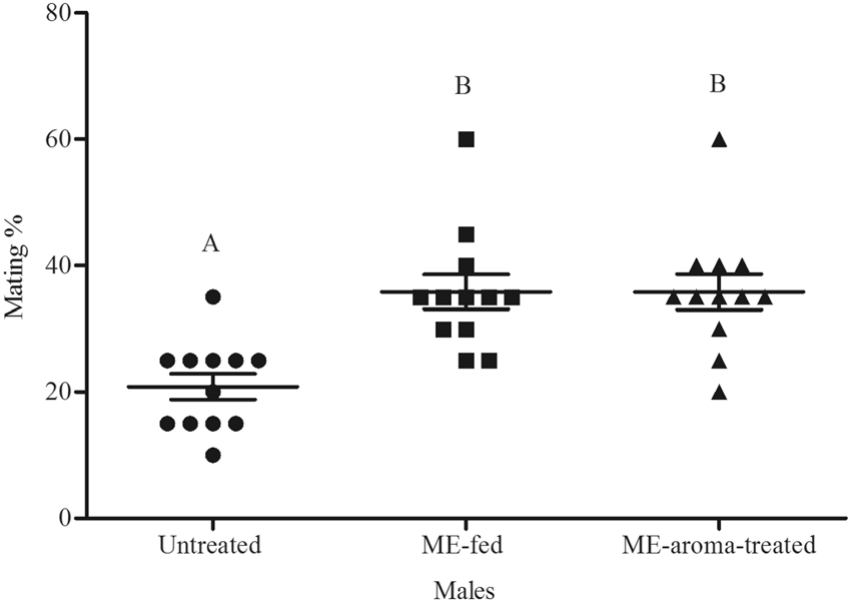
Mean mating percentage of ME-fed, ME-aroma-treated, and untreated *Bactrocera dorsalis* males. The age of males on commencement of mating test was 16 d and they were treated with ME 2 d before mating test. Symbols represent raw data for 12 replicates; horizontal lines represent mean ± SE. Mean male mating success followed by different letters are significantly different from each other (Tukey’s Test, *P* < 0.05).

**Figure 6:**
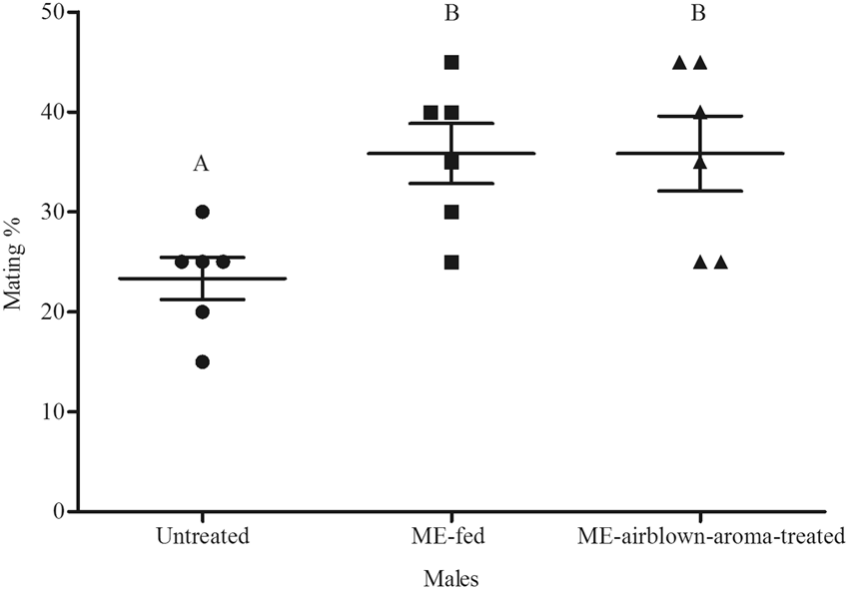
Mean mating percentage of ME-fed, ME-airblown-aroma-treated, and untreated *Bactrocera dorsalis* males. The age of males on commencement of mating test was 15 d and they were treated with ME 2 d before mating test. Symbols represent raw data for 12 replicates; horizontal lines represent mean ± SE. Mean male mating success followed by different letters are significantly different from each other (Tukey’s Test, *P* < 0.05).

In experiment 1, ME-aroma-treated males competed with untreated males, and in experiment 2, ME-airblown-aroma-treated males competed with untreated males. In both experiments, the treated males achieved significantly higher mating success (Figs 1 and 2 respectively) than untreated males (*t* = 2.52, df = 18, *P* < 0.05 and *t* = 4.41, df = 18, *P* < 0.001, respectively). In Experiment 3, ME-aroma-treated males achieved similar mating success as compared with ME-fed males (*t* = 0.87, df = 6, *P* > 0.05; Fig. 3). In experiment 4, ME-airblown-aroma-treated males achieved similar mating success as by ME-fed males (*t* = 0, df = 6, *P* > 0.05; Fig. 4). In experiment 5, testing ME-fed, ME-aroma-treated and untreated males, the male mating success was significantly different among the different treatments (*F*_1,35_ = 11.53, *P* < 0.001). Mating success of ME-fed and ME-aroma-treated males was similar but significantly higher (Tukey’s Test, *P* < 0.05) than that observed for untreated males (Fig. 5). Similarly, in experiment 6 where ME-fed males were competing with ME-airblown-aroma-treated and untreated males, the mating success was also significantly different among the differently treated males (*F*_1,17_ = 5.68, *P* = 0.014). Mating success between ME-fed and ME-airblown-aroma-treated males was similar, and the mating success of both type of treated males was significantly higher (Tukey’s Test, *P* < 0.05) than that observed for untreated males (Fig. 6).

## Discussion

These studies showed that sexually mature *B*. *dorsalis* males treated with ME-aromatherapy, ME-airblown-aromatherapy, and ME-feeding achieved similar levels of mating success, and the mating competitiveness of all ME-treated males was significantly higher than that of untreated males. In separate comparisons involving ME-aroma-treated vs. untreated males, the treated males achieved significantly more matings than untreated males.

ME feeding is known to enhance mating success of males of several *Bactrocera* species, including *B*. *dorsalis*^6^ and after feeding, ME is enzymatically transformed into other metabolites which are released in the male pheromone^27,39,40^. The males obtain their mating advantage due to the greater attraction of females to these metabolites^27,41–43^. Therefore, it was assumed that only ME feeding could enhance the mating success of the male flies. *Bactrocera dorsalis* males exposed to ME but with restricted contact (ME covered by a screen mesh) had no mating advantage according to Shelly and Dewire^25^. However, our results clearly indicated that ME application by aromatherapy (males had no physical contact with ME source) did enhance *B*. *dorsalis* male’s mating success in the same way as ME feeding. The discrepancy between the two studies could be related to the duration of males staying on the screen-top of the petri-dish containing the ME, i.e. 2 h exposure in the first study^25^ and up to 5 h in our study, or until they started to leave the petri-dish. Although experiments testing different durations for male’s exposure to ME for their effects on mating success were not conducted in the current study, it seems that exposing males until they achieve satiation is critical. This was also evident from the previous study with *B*. *carambolae*, where males started to leave the screen top petri-dish containing ME after ca. 3 h^38^, while in the current study, which maintained all other conditions, *B*. *dorsalis* males started leaving the petri-dish after 4–5 h. The difference in required ME exposure duration of *B*. *carambolae* and *B*. *dorsalis* could be related to differences in required dose of ME to get the mating advantage, which is much lower for *B*. *carambolae* males (0.18 μl) as compared with *B*. *dorsalis* (0.70 μl)^44^.

For the SIT application, ME exposure by means of feeding would bring significant advantages in terms of enhanced sterile male mating success and low incidence of repeat feeding in the field after release. This would greatly enhance the cost effectiveness of the SIT as compared to the current scenario where males are not treated with ME before release. However, ME feeding appears to cause over-consumption inducing toxicity^32^ that temporarily reduces the males’ agility^25,38^. Shelly and Dewire^28^ demonstrated that males fed on ME for 2 h achieved less matings than untreated males on the day of feeding, but such treated males got a mating advantage 2 d after feeding. However, males fed on ME only for 30 seconds gained a mating advantage on the same day of feeding. During the course of experiments we noticed that ME feeding caused at least 10–15% mortality of males, but mortality was very rarely observed in ME-aroma-treated males. Considering a scenario where millions of sterile males will have to be treated on a weekly basis, this considerable mortality will represent a significant increase in cost for operational SIT programmes. Therefore, a ME delivery system that could provide a precise quantity of ME by allowing sterile males to obtain ME until satiation without causing mortality or collateral effects on fly quality is highly desirable. Unlike feeding of ME, its application by aromatherapy or airblown-aromatherapy seems to give males the control to continue acquiring the ME until achieving satiation. This delivery of the ME could be easily adjusted and fine-tuned by determining the proportion of ME that should be released per m^3^ in a given period of time and for a given number of sterile males.

The results presented in the current study clearly showed that ME aromatherapy and airblown aromatherapy, analogous to ME feeding, enhanced mating success of *B*. *dorsalis* males. This however, begs the question of how aromatherapy and direct feeding can have a similar effect on male mating success. It is conceivable that males exposed to aromatherapy and airblown aromatherapy somehow acquire volatiles of ME (including the possibility that they become absorbed through body surface, including the pumping by the proboscis). These are then processed using a similar pathway for pheromone production as after ME feeding (pheromonal effect) and/or may somehow trigger the accelerated metabolic rate of males. However, chemical analysis of rectal glands and pheromone volatiles, as has been done for ME-fed males, would be needed to provide the answers on how ME-aromatherapy enhances the male mating success.

Our previous work^38^ and the data presented in this paper have clearly shown that, independent of the specific mechanism that allows ME intake, delivery of ME by aromatherapy enhances the mating success of *B*. *carambolae* and *B*. *dorsalis* males. As a next step, the development of a practical ME delivery system was needed that could be adopted in fly emergence and release facilities that have to process millions of sterile males each day. A prototype for an airblown-ME delivery system was developed that permitted the males contact with the ME volatiles, while prohibiting their direct feeding. Our airblown-aromatherapy system is similar to the GRO aromatherapy system used in sterile male’s release facilities for SIT application for management of *C*. *capitata*^29^. We have shown in our study that our system of blowing air can deliver the required amount of ME volatiles, but this design will of course need to be improved and up-scaled for practical use in operational programmes.

Enhanced mating competitiveness of *Bactrocera* species following ME exposure will reduce the numbers of sterile males that need to be released, which will reduce the cost of SIT application^45^. Since it has been estimated in field enclosure tests that 84% less sterile ME-treated males would be needed than non-exposed males to induce a similar level of sterility in females^46^, this lower number of sterile male requirements would reduce the cost of SIT application significantly. However, the response of ME-airblown-aroma-treated males to repeat feeding on ME still needs to be assessed in the field to check whether ME-airblown-aroma treatment suppresses sufficiently male’s re-visits to ME as it was done for ME-feeding, e.g. ME-fed males were two to three times less responsive to re-visiting ME source^21^. Having a viable aromatherapy for *B*. *dorsalis* would have a huge economic impact on area-wide management programmes for *Bactrocera* fruit flies, as fewer sterile males would be needed in order to cover larger horticultural production areas.

## Material and Methods

### Study insects

The *B*. *dorsalis* flies used in this study originated from the genetic sexing strain (GSS) developed at the University of Hawaii, USA^47^. This GSS is characterised by a pupal colour mutation in which a wild-type copy of the marker is attached to the Y-chromosome so that males express the wild phenotype (brown pupae) and the females express the mutant phenotype (white pupae). The sexes can be separated at the pupal stage with a colour sorter, males only can be released in the target area, a strategy known to increase the effectiveness of the SIT application against *C*. *capitata*^48^. Since its development in 1995^47^, the strain has been maintained at the FAO/IAEA Insect Pest Control Laboratory in Seibersdorf, Austria. The larvae of the flies were maintained on a carrot powder-based diet that was modified from the standard, wheat bran-based diet^49^. Following emergence, the adult flies were provided with standard adult diet containing sugar and hydrolyzed yeast in a 3:1 ratio and water *ad libitum*. Both sexes were separated using the colour sorter at the pupal stage and maintained separately at 24 ± 1 °C, 60 ± 5% RH and a photoperiod of 14L:10D (initiation of darkness period was adjusted in accordance with sunset time) in plexiglass tubular cages (45 cm × 20 cm diameter) having both openings covered with cloth mesh. The photoperiod in laboratory was adjusted according to the natural photoperiod, thus males were ready for mating activities when released at dusk time. The flies used in a given experiment were from the same batch of pupae. Males were marked on the thorax with different colours of a water-based paint before maturation and used in the different treatments. Males of all treatments were held separately from females.

### Treatments

#### ME-feeding

**Figure 7:**
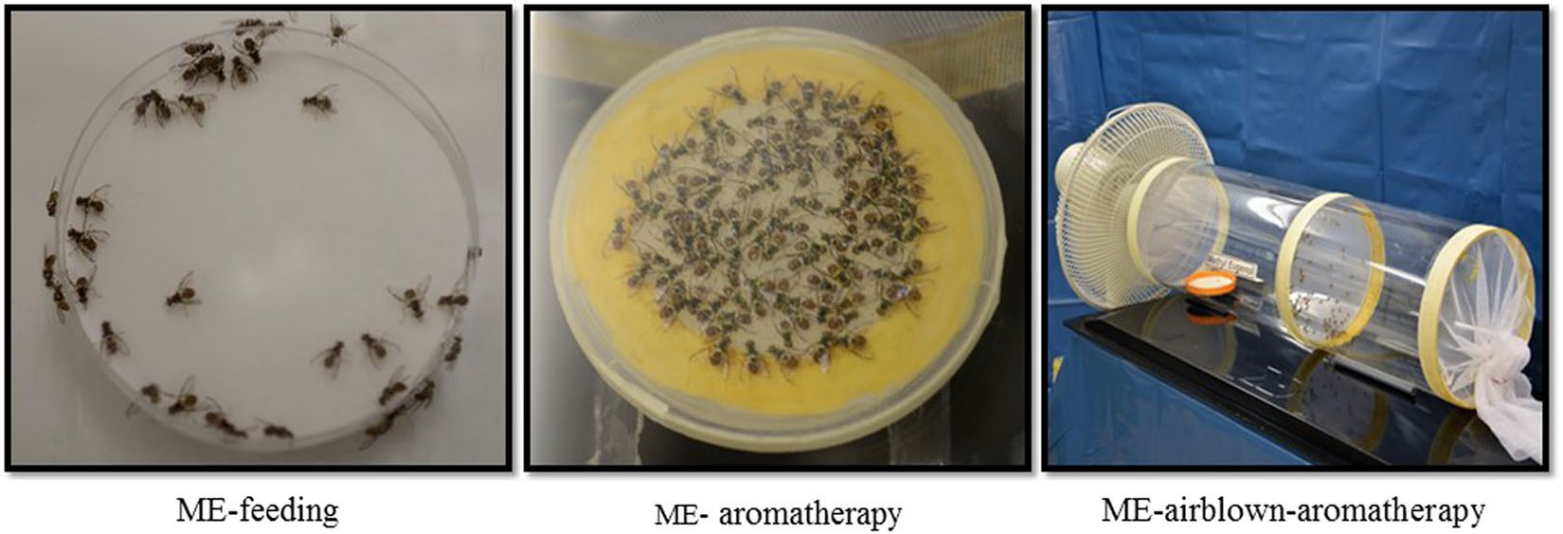
Three different ME delivery systems were applied to *Bactrocera dorsalis* males. The ME-aromatherapy design excluded the possibility of male’s direct contact with ME source and feeding on ME.

ME-feeding was carried out in a room isolated from the fly cultures. One d before feeding, males were immobilized by holding them under netting and marked on the thorax with a dot of water-based paint. Marked males (13–14 d old, n = 120) were transferred to a plexiglass tubular cage (20 cm × 20 cm diameter) that had both openings covered with cloth mesh. ME (0.5 ml) was placed on a filter paper strip, which was then placed in a petri-dish (6 cm diameter) and introduced into the cage (Fig. 7). Marked males (13–15 d old) were allowed to feed on the ME (hereafter called ME-fed males) for 1 h between 09:30–10:30 h which corresponds to the peak ME foraging time^50^. The feeding activity of individual males was not monitored during the 1 h exposure period. The petri-dish containing the ME was then removed, and the treated filter paper strip was sealed in a polythene bag and discarded. The males were provided with standard adult diet and water *ad libitum*. ME feeding is reported to enhance mating success of *B*. *dorsalis* males 1 d after feeding, and this effect remains visible as long as 35 days after feeding^25,27^. Therefore, ME-treated males were evaluated 1 or 2 d after ME treatment, and when tested males were 15–17 d old.

#### ME-aromatherapy

ME-aromatherapy was carried out in another room isolated from the fly culture room and the ME-feeding room. The male flies were marked 1 d before treatment as described above. Marked males (13–14 d old, n = 100) were transferred to a plexiglass tubular cage (20 cm × 20 cm diameter) that had both openings covered with cloth mesh. ME (0.5 ml) was introduced in the same manner as described above, except that the petri-dish was covered with fine nylon mesh that prevented the males having contact with the ME source (Fig. 7). Marked males (13–15 d old) were exposed to ME volatiles (hereafter called ME-aroma-treated males) for 5 h (09:30–14:30 h). The males started to move away from the petri-dish after 4–5 h, and slight shaking of petri-dish resulted in remaining males moving also away. The petri-dish was then removed, and the treated filter paper strip was sealed in a polythene bag and discarded. After ME treatment the males were provided with standard adult diet and water *ad libitum*. The males were tested 1 or 2 d after ME exposure when males were 15–17 d old.

#### ME-airblown-aromatherapy

Haq *et al*.^38^ demonstrated ME-aromatherapy enhanced the mating success of *B*. *carambolae* males, but the exposure method was not conducive to large scale exposure that would be required in fruit fly emergence and release facilities. Therefore, we designed an airblown system for ME application (hereafter called ME-airblown-aroma-treated males) that was similar to ME-aromatherpay (described above) except that ME was delivered by means of blowing air. Marked males (13–15 d old) were exposed to ME by keeping them at least 45 cm away from an ME source by a plexiglass tubular cage 45 cm long and 20 cm diameter covered with cloth mesh at one opening, and the other opening was uncovered. A cage of similar size holding males and having both openings covered with cloth mesh was placed connected to the open end of the tube containing the ME. A small table fan (model DT 630E; http://de.ventilator.org/duracraft/dt-630e/) placed at the covered opening of the tube was used to blow air through the ME containing tube. This set up dispensed ME volatiles to males by blowing air for 5 h (Fig. 7). The males were tested 1 or 2 d after ME exposure at an age of 15–17 d.

#### No-ME treatment

Male flies that were not exposed to any ME treatment (hereafter called untreated males) were maintained on standard adult diet and water *ad libitum* in another room isolated from the rooms used for ME-feeding or ME-aromatherapy. Untreated males were marked on the same day as the treated males and maintained in cages in the same manner as the treated males. When tested, untreated males were 15–16 d old.

### Mating competitiveness tests under field cage conditions

#### Field Cages

The walk-in field cages used for mating trials were screened, circular tents (2.2 m high × 2 m diameter)^51^, each containing a potted citrus tree of 2 m height. Eight such field cages were placed inside a large insect greenhouse (24 m × 10 m × 4 m) that allowed us to carry out eight replicates of the test simultaneously. A temperature of 26 ± 2 °C and 60 ± 5% RH was maintained through-out the experiment. The insect greenhouse had a translucent roof that provided semi-natural illumination.

#### Experiments under Field Cage conditions

Experiment 1. Mating competitiveness of ME-aroma-treated males against untreated males: Twenty ME-aroma-treated males and 20 untreated males were released simultaneously in a field cage approximately 90 minutes before sunset to allow them to acclimatise with their surroundings before initiating their mating activities. *B*. *dorsalis* males are reported to initiate mating activities by calling and releasing pheromones approximately 90 minutes before sunset^52^, therefore, a similar window of time was selected for the present trials. Fifteen minutes after male release, we introduced 20 virgin untreated females (same age as that of males) into the field cages. As soon as mating occurred, the pairs were carefully collected separately in each vial. Experiments were concluded one hour after sunset. Artificial illumination (white light) was used to detect and collect any remaining mating pair in the dark. The pairs were brought in vials to the laboratory for identification. A maximum of five replicates per day and total of ten replicates were carried out for this experiment.

Experiment 2. Mating competitiveness of ME-airblown-aroma-treated males against untreated males: The same protocol used in experiment 1 was followed, except that in this experiment only ME-airblown-aroma-treated males were competing with untreated males. A maximum of five replicates per day and total of ten replicates were also carried out for this experiment.

Experiment 3. Mating competitiveness of ME-aroma-treated males against ME-fed males: Procedures were the same as in experiment 1, except that ME-aroma-treated males were competing against ME-fed males and four replicates were carried out simultaneously for this experiment.

Experiment 4. Mating competitiveness of ME-airblown-aroma-treated males against ME-fed males: The same procedure used in experiment 1 was followed, except that in this experiment ME-airblown-aroma-treated males were competing with only ME-fed males, and four replicates were carried out simultaneously for this experiment.

Experiment 5. Mating competitiveness of ME-aroma-treated males against ME-fed males and untreated males: The experimental protocol as in experiment 1 was followed, but ME-fed males, ME-aroma-treated males and untreated males were competing for mating with untreated virgin females. A maximum of four replicates per day and total of twelve replicates were carried out for this experiment.

Experiment 6. Mating competitiveness of ME-airblown-aroma-treated males against ME-fed males and untreated males: The experimental protocol as in experiment 1 was followed, but ME-fed males, ME-airblown-aroma-treated males and untreated males were competing for mating with untreated virgin females. A maximum of four replicates per day and total of six replicates were carried out for this experiment.

### Data analyses

Data expressed as relative mating success (% of total possible matings) by ME-aroma-treated/ME-airblown-aroma-treated males vs. untreated males and ME-fed vs. ME-aroma-treated/ME-airblown-aroma-treated males fulfilled parametric assumptions (data were normally distributed) and were analysed by the unpaired two-tailed Student’s *t*-test^53^. Similarly, the expressed data for mating success by ME-fed, ME-aroma-treated/ME-airblown-aroma-treated males, and untreated males in experiment 5 and 6 also fulfilled parametric assumptions (data were normally distributed) and were analysed by One-Way ANOVA^53^. The significance value used in data analysis was 95% α ≤ 0.05). Complementary pair-wise comparisons of means were performed by Tukey’s test^53^.
